# Colloidal photonic crystals formation studied by real-time light diffraction

**DOI:** 10.1515/nanoph-2022-0127

**Published:** 2022-06-09

**Authors:** Jose Ángel Pariente, Álvaro Blanco, Cefe López

**Affiliations:** Consejo Superior de Investigaciones Científicas (CSIC), Instituto de Ciencia de Materiales de Madrid (ICMM), Calle Sor Juana Inés de la Cruz 3, E-28049 Madrid, Spain

**Keywords:** colloidal crystallization, *in-situ* characterization, nanophotonics, self-assembly

## Abstract

Colloidal suspensions crystallize by a natural sedimentation process under certain conditions, the initial volume fraction being one of the parameters that govern this process. Here, we have developed a simple *in-situ*, real-time, optical characterization technique to study silica colloidal suspensions during natural sedimentation in order to shed new light on this crystallization process. This technique monitors small variations in the wavelength of the reflectance features, allowing the analysis of the formation of the first layers of the crystal with sub-nanometer precision, and their dynamics, which is crucial to ensure a high quality in the final sample. The experimental results indicate that, in certain range of volume fraction, spontaneous crystallization of a colloidal fluid occurs at the bottom of the suspension, as a phase change, then through evaporation of the water it compacts to near close-packed and, eventually, dries. Understanding self-assembly at these scales is paramount in materials science and our results will contribute to improve and characterize the quality and crystallinity of the materials used in this process.

## 1 Introduction

Assemblies of particles in nature have been widely studied as a simple model for the behavior of condensed matter in its various states. Since colloidal particles exhibit Brownian motion and, at the same time, are large enough to trace their assembly [1], the analogy with atomic crystallization is one of the most recurrent examples in literature [2, 3]. Freezing and melting during this process enables the study of phase transitions [4, 5], where there are a multitude of physical mechanisms involved whose analysis is in great demand. As evidence, the phenomenon underlying spontaneous crystallization, entropy [6], has not been dilucidated until less than two decades ago [7].

Here, among many other self-assembly techniques [8], natural sedimentation [9] emerges as an excellent method, that enables the analysis of diverse phenomena during particle settling [10–12] such as dynamics [13, 14], self-diffusion coefficient [15] or crystallization [16, 17]. At variance with the most widely used technique, convective self-assembly, in the natural sedimentation method the growth direction is perpendicular to the sample plane allowing to study the assembly. Therefore, a “simple” sedimentation process provides invaluable information regarding these parameters in a highly non-ideal system, all of which is supported by abundant theoretical work [18–20].

A much less studied process that has a critical influence on the final arrangement of the spheres is the evaporation of the solvent [21]. Probably, the reason behind is that, in some self-assembly methods, the drying process and the formation of the crystal usually proceed simultaneously [22, 23]. Therefore, water inspection has been mainly performed by its adsorption onto dried colloidal crystals [24], or by optical microscopy during the drying process [25, 26]. While studies agree on the morphology adopted by water during evaporation which, in the final stages, forms “necks” between the spheres [27], an *in situ* characterization of the problem could shed additional light on this phenomenon.

Given the importance of this assembly mechanism, numerous techniques have been employed for its precise characterization such as confocal microscopy [28], X-ray diffraction [29, 30] or light scattering [31], to name a few. Other studies have used Bragg diffraction, specifically interference of Fabry–Perot fringes, to monitor crystal growth in suspensions of different salt concentration [32], while others have employed the same phenomenon to observe the evolution of the volume fraction at different length scales during the evaporation process [33]. In colloidal suspensions, the sedimentation profile is basically barometric and only when the particle concentration increases considerably, the interactions between the particles change the profile significantly. In our study, we start with very dilute suspensions that concentrate towards the bottom of the vial as the sedimentation process evolves. This concentration evolution can be monitored in real time, which helps us to understand the crystallization process. In this way, the phase transition, marked by the spontaneous crystallization of the colloidal fluid, is observed by the sudden emergence of the Bragg peak. This provides an accurate analysis of the self-assembly of the first layers of the crystal and their further growth by tracking this peak which reveals real time light diffraction as a useful technique to trace the organization of the spheres during the natural sedimentation process. The latter is critical in the fabrication of self-assembled materials to optimize the arrangement of their building blocks.

## 2 Experimental section

### 2.1 Colloids

Colloidal suspensions of commercial silica particles (microParticles GmbH) with diameter size, *d* = 250 nm (polydispersity below 3%), and different volume fractions were prepared in the same colloidal volume (*V*_0_ = 4 mL) to obtain the range of concentrations that result in the crystallization of the settled structures. Particles were used as purchased without further purification or functionalization. To ensure homogenization of the colloidal suspensions, they were first immersed in an ultrasonic bath for 30 min to remove any particles stuck to the tube walls, and then they were redispersed for 2 min.

### 2.2 Sedimentation

**Figure 1:**
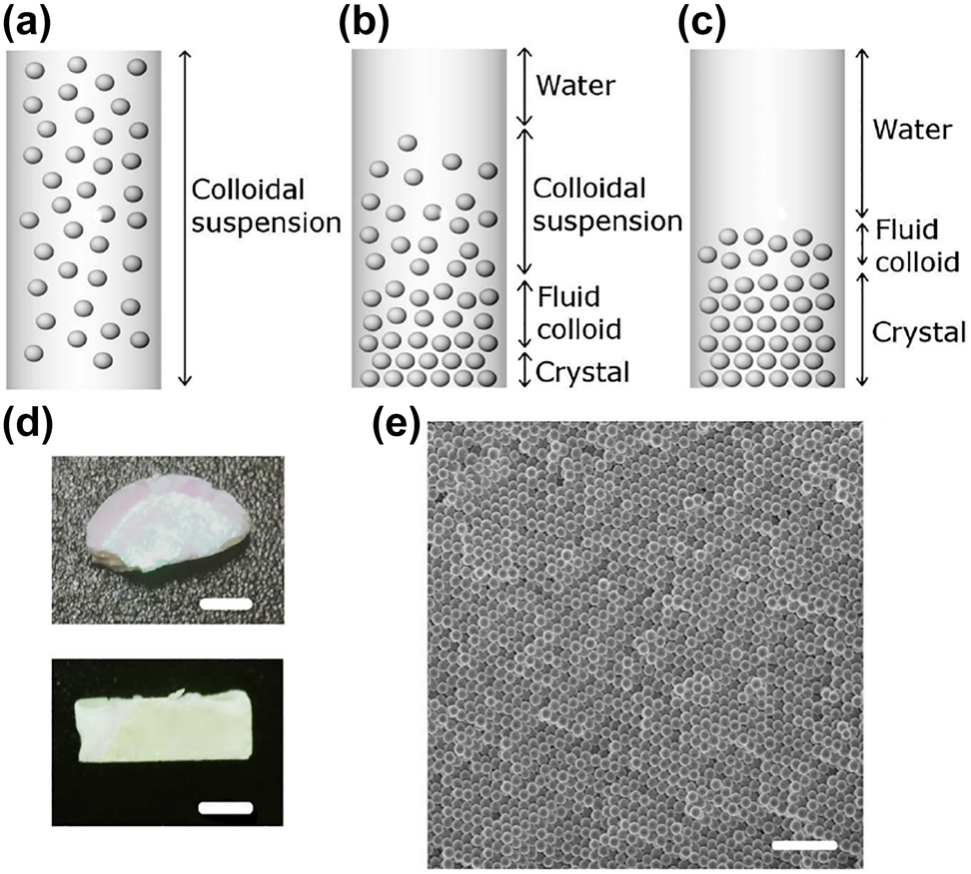
Schematic illustration of a colloidal suspension of silica spheres in different phases: initial (a), during (b) and final (c). (d) Optical image of a dried thick sample with a *ϕ*_0_ = 0.037. (e) Scanning electron microscope (SEM) image of a sample with *d* = 250 nm and *ϕ*_0_ = 0.018 that shows an *fcc* lattice. The scale bars are 3 mm (d) and 1.25 µm (e).

A polypropylene tube, 1 cm^2^ cross section and 5 cm height, placed on a glass slide that is covered to prevent evaporation in the first stages of sedimentation, is used as a container for the colloids where the spheres sediment (Figure 1a, b). The hydrophobic inner surface of these polypropylene tubes reduces particle binding to the walls. Colloidal suspensions are prepared in water and no salt is added to avoid disturbing the self-assembly of the spheres [34]. Once the spheres have settled (Figure 1c), a volume of 2 mL of water is carefully extracted in order to expedite the water evaporation process that would, otherwise, take many days to complete. This is a safe volume of water that can be removed without disturbing the remaining colloidal suspension.

### 2.3 Drying

From then on, all the water evaporates under ambient conditions, 25 °C temperature and 35% relative humidity. The ambient conditions of all experiments are quite similar since the temperature of the room is controlled. Normally, the colloidal crystals obtained are thicker (Figure 1d) than those obtained by other methods and, obviously, their final thickness depends on the concentration used in the initial suspension.

### 2.4 Electron microscopy

Analysis of the scanning electron microscopy (SEM) images of the silica spheres made it possible to estimate the diameters of the spheres at *d* = 250 nm, with a 5% of polydispersity. Figure 1e shows an SEM image of the upper (free) surface of a sample prepared with an initial volume fraction *ϕ*_0_ = 0.018 of *d* = 250 nm spheres.

### 2.5 Optical characterization

Light diffraction by the crystalline structure was measured as a function of time to characterize the packing of the spheres as they settle. Reflected light is collected at normal incidence by an optical fiber that is also used to illuminate the underneath part of a glass slide with a collimated beam creating a 2 mm diameter spot (see SI and Figure S1). As a result, the crystal structure was observed from the bottom of the cell. By means of real-time diffraction, the characterization of the particles assembly is carried out directly in this type of systems while they evolve during sedimentation.

## 3 Results and discussion

To trace the organization of the spheres, we used a natural sedimentation set up where colloids of controlled initial volume fraction were poured in sedimentation tubes through whose transparent substrate; the reflectance of the forming crystal was measured by standard spectroscopic techniques. Figure 1 shows a schematic of the colloid evolution and the resulting product. The evolution from a loose to a close packed structure in a sedimentation process is described by the volume fraction, *ϕ*. This parameter is the only one that can change as the spheres settle since the refractive index of the spheres and that of the medium remain constant. Since both average refractive index and lattice parameter are directly tied to *ϕ*, the Brag wavelength can be expressed as a function of the volume fraction [35]:

(1) $$\lambda =d\frac{2}{\sqrt{3}}{\left(\frac{2\pi }{3\phi }\right)}^{1/3}{\left[\phi n_{p}^{2}+\left(1-\phi \right)n_{m}^{2}\right]}^{1/2}$$

where *λ* is the wavelength, *d* is the diameter of the spheres, *n*_p_ is the refractive index of the particles and *n*_m_ is the refractive index of the medium. For the sake of efficiency, rather than solving for *ϕ*, it is useful to fit the above expression to a power law, with the following result:

(2) $$\phi =\frac{10^{9}}{\lambda^{3.3}}$$

The regression of the fit is excellent and the fit produces error of less than 0.1% so it will be used to calculate the volume fraction of the colloidal crystal and, from the analysis of the different spectra taken as a function of time, its temporal evolution will be obtained.

**Figure 2:**
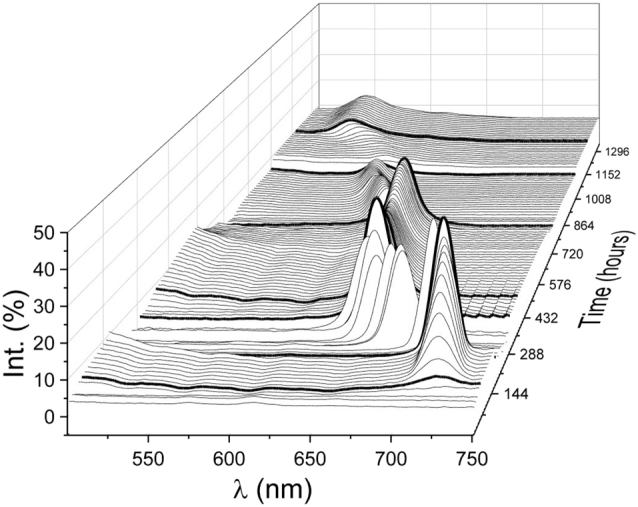
Specular reflectance signal monitoring the formation of the Bragg peak and its evolution as a function of time. The initial hundred hours or so have been removed from the plot to show only the range of interest.

The results obtained in this experiment provide, on the one hand, boundaries to initial conditions of preparation of colloidal crystals by natural sedimentation and, on the other, describe the crystallization dynamics when such conditions lead to the crystalline phase. Additionally, they provide means to estimate the thickness of the grown crystal in real time. A typical result is reported in Figure 2 for a case in which crystallization actually happens. Several alterations in spectral positions, intensities and background take place with different rates of change along the whole process which require the use of different time scales to properly visualize them. These spectra are divided into different figures (see SI and Figure S2) to clarify this behavior.

At the beginning of the process, the measured spectra only present a constant background of reflected light. After about one hundred hours, the first hints of the Bragg peak due to the periodicity of the spheres deposited on the bottom of the suspension can be observed (thick black line). The peak grows and blue shifts as spheres disappear from the colloidal suspension by the continuous sedimentation, until it eventually stops and stabilizes. At this point, the excess liquid is manually removed and the tube is opened, so that water evaporation begins. A further blue shift of the Bragg peak is again monitored, when the air–water interface reaches the interface between supernatant water and colloidal fluid, due to a decrease in the volume available for the spheres. This process continues until the structure can no longer be compressed, at which point, evaporation begins extracting water from the pores of the crystal. The consequent change in the refractive index contrast of the crystal results in a rapid shift of the peak until this is stabilized by drying out almost all the water in the structure.

**Figure 3:**
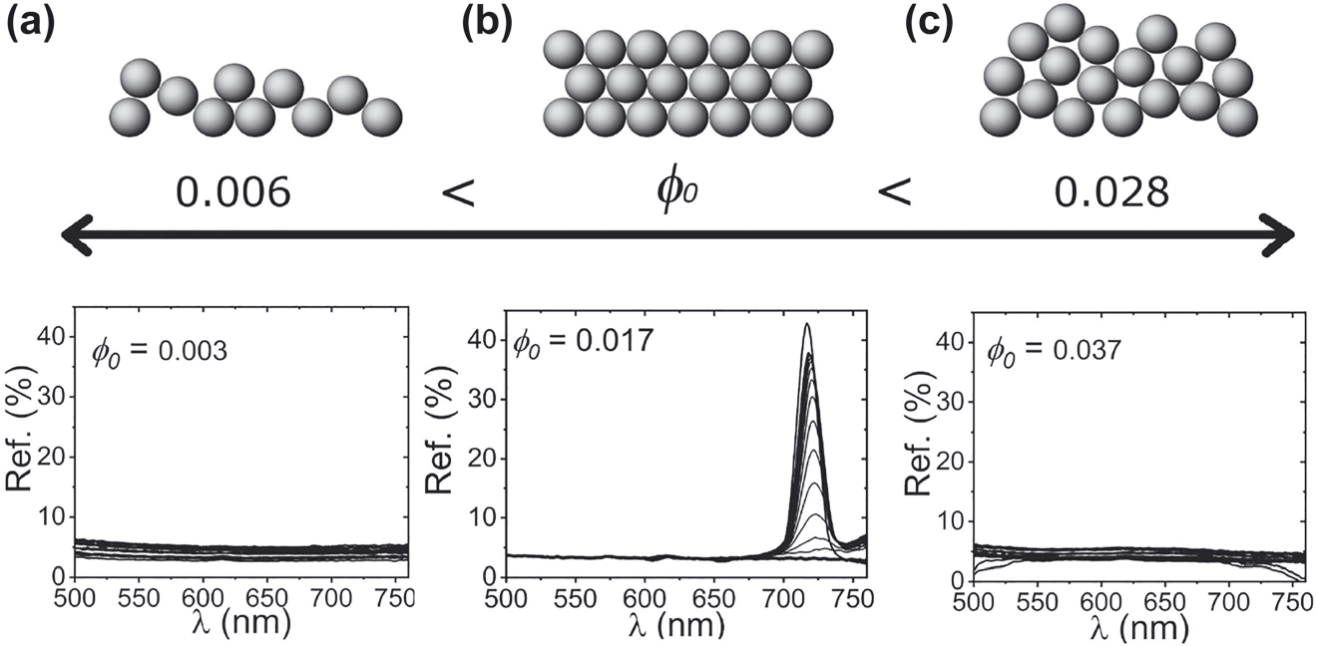
Phase diagram of our system with *d* = 250 nm where the fluid (a), crystalline (b) and amorphous (c) phases can be observed. The formation of a crystalline phase from a colloidal suspension is achieved for a certain initial volume fraction, *ϕ*_0,_ range whose bounds are exposed by the Bragg peak emergence and disappearance.

The control parameter chose to follow the self-assembly process is the spheres volume fraction, *ϕ*_0_, of the initial suspension. Other parameters such as the sample height [36] or the subsequent solvent evaporation rate [37], could also have an impact on the final arrangement of the structure although, for clarity, we limit our study to a single parameter. The influence of the initial volume fraction can be seen in Figure 3 where three different ranges are shown and illustrated by successive reflectance spectra. The limits of the initial volume fractions were determined by testing different *ϕ*_0_ and observing which of them crystallized and which did not.

For low initial concentration, *ϕ*_0_ < 0.006, the reflection of the sample slowly increases during sedimentation and no peak is observed throughout the process. This is shown in Figure 3a where the concentration of particles is too low for the transition to the crystalline phase to occur, so that these suspensions remain in the colloidal fluid phase indefinitely [14]. Nevertheless, even at this point, the crystallization of the colloidal fluid can be induced once sedimentation is completed by adding a certain volume of spheres that causes the equivalent initial volume fraction to increase over the threshold *ϕ*_0_ > 0.006. In order to do this, a calculated amount of more concentrated colloid is carefully pipetted in on the clear water at the top of the vial so that the final concentration is brought above the threshold (*ϕ*_0_ > 0.006). This is shown in Figure S3 where the specular reflectance from a colloidal suspension with *ϕ*_0_ = 0.005 is monitored for 550 h (the sedimentation of all the spheres would not take more than 385 h). At *t* = 599 h (thick black line), a supplementary *V* = 0.3 mL (*ϕ*_0_ = 0.011) of solution of the same spheres is added to the colloid resulting in an equivalent volume fraction of *ϕ*_E_ = 0.006, thus above the threshold of the phase diagram. In just a few hours, the newly added spheres settle and cause the colloidal fluid deposited at the bottom to crystallize, as marked by the appearance of the Bragg peak as seen in Figure S3.

When 0.006 < *ϕ*_0_ < 0.028, the organization of the spheres at the bottom of the suspension occurs and is revealed by Bragg diffraction. Its onset signals the transition to the crystalline phase with the emergence of a peak in the reflectance spectra, Figure 3b, caused by the periodicity in the assembly of the spheres.

If the initial volume fraction is too high, sedimentation leads to a disordered phase too. Here, pressure is too high hampering particles mobility impeding crystallization. This process gives results similar to attempts to crystallization under centrifugation [38]. Figure 3c shows the results for *ϕ*_0_ > 0.028 where particle diffusion in high concentration samples is hindered to the point that no crystals are formed in this timescale, even though crystallization is still preferable in terms of thermodynamics [4]. The suspensions remain in a metastable amorphous phase that could crystallize for a certain height, where the spheres can diffuse by a reduction in the osmotic pressure of the colloid as has already been observed in previous studies [16]. Crystallization has been observed in this range in special conditions including microgravity [39].

The iridescence shown in Figure 1d, visible only on the top side of the colloidal compact, illustrates the fact that, samples prepared outside the concentration range for crystallization can acquire certain order in a number of layers. This portion is crystallized owing to strong capillary forces experienced at the drying stages of the evaporation when only the top layers’ spheres retain some mobility, the bottom of the sample remaining locked. In addition, the osmotic pressure in the natural sedimentation depends on height in the sediment [1], and therefore, also *ϕ*, causing the spheres to diffuse more easily in the upper part of the structure. However, these phenomena are not detected by our experimental set-up that inspects the samples from below.

**Figure 4:**
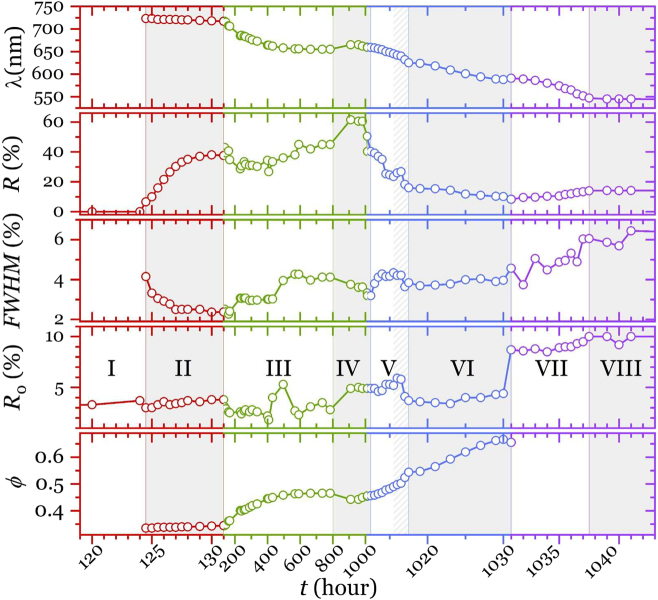
Optical monitoring. Features from the reflectance spectra as a function of sedimentation time in hours for *ϕ*_0_ = 0.017 comprising Bragg wavelength, peak reflectance, background reflectance and volume fraction from Eq. (2). Time has been divided in four epochs (red, green, blue and violet axes) where the scale is chosen attending to the level of detail required to show the phenomena taking place. Shade areas mark the alternating regions as explained in the text.

Once the conditions leading to crystallization at the bottom of the vessel (0.006 < *ϕ*_0_ < 0.028) are established, we describe its dynamics. For this purpose, we study the optical properties of the assembled crystal from which volume fraction can be derived. The evolution of the Bragg peak is recorded as a function of sedimentation time as exemplified in Figure 4. For the sake of clarity, real-time light diffraction is described for *ϕ*_0_ = 0.017 although such behavior is similar for all other concentrations. The sedimentation and drying can be divided in several stages (marked as alternating clear and shaded regions) whose precise time boundaries vary depending on the sphere size and the initial volume fraction used in the colloid preparation. The different time regions of the sedimentation are summarized in Table 1 where the processes taking place and the events at which each period stars and finishes are also noted.

**Table 1:** Time zones describing the onset and final state and the process taking place.

Region	Begins when	Process	Ends when
I	Pour colloid in vessel	Brownian motion & gravity sedimentation	Amorphous sediment forms
II	First layers crystallize from amorphous	Nucleation and growth	Crystallized sediment and fluid coexist
III	Thick crystal formed	Crystal compaction	Compressed crystal reaches equilibrium
IV	Pipetting and uncovering vessel	Water evaporation	Clear water exhausted
V	Water level reaches colloidal fluid	Volume shrinking compression. Freezing (*ϕ* = 0.495)	Melting (*ϕ* = 0.545)
VI	Fraction precludes fluid	Evaporation draws water from crystal	Near close-packed crystal in water
VII	Water reaches crystal edge	Interstitial water evaporation	Humid close packed silica@air crystal
VIII	Free water inside crystal exhausted	Adsorbed water ceases to evaporate	Dry crystal

### 3.1 Colloid sedimentation *(0–124 h)*

Many hours after the colloid is poured in the cylinder (Figure 1a), a dense sediment is formed at the bottom of the tube at the expense of particles depleting the top region of the colloidal suspension where a region of clear water is left; its height is determined by the Stokes velocity. Up to this point no special optical features are observed except for a uniform background of diffuse light reflected by the colloidal suspension and the substrate itself. This can be appreciated in Figure 4, region I where the signal is just background where no special wavelength can be identified.

### 3.2 Nucleation and crystallization growth *(124–131 h)*

Depending on initial volume fraction, at a certain time a Bragg peak associated to the band gap starts to form which signals the transition from the fluid colloid to the crystalline phase at the bottom of the sedimentation cell (Figure 1b). As the crystal forms, it grows by adding new layers (hence increasing Bragg peak intensity and reducing peak width). This process is very sudden in relative terms as it lasts only a few hours as can be seen in Figure 4, region II. It should be noted that the entire colloid deposited at the bottom does not crystallize immediately, but only the spheres closest to the substrate undergo this transition. The profile of the volume fraction of spheres in the sediment depends on height [10], causing a gradient in the gravitational pressure of the sediment, resulting that only the spheres at the bottom crystallize (greater pressure) [1]. However, the spheres located far from the bottom do not undergo this transition and therefore remain in the colloidal fluid phase. This colloidal fluid crystallizes as more spheres are deposited, causing both phases to coexist in the sediment (Figure 1b). The detection time, however, varies with initial concentration so that larger concentrations lead to earlier nucleation, as shown in Figure S4. This can be understood as the higher the initial colloidal concentration, the sooner sufficient spheres reach the bottom just because more spheres are close to it.

Based on the reduced breadth of the Bragg peak it can be assumed that diffraction is caused by the formation of a large crystalline domain. Multiple crystal orientations occurring at random would, at best, lead to a broad peak average of many random diffraction configurations governed by the Bragg law, $m\lambda =2\bar{n}d\;\cos\;\theta$ . It is safe to assume that such dispersion is larger than the peak width governed essentially by the small contrast between water and silica refractive indices [40]. A simple calculation based on scalar wave approximation, SWA [41], predicts Δ*ω/ω* ≈ 2% for the relative *FWHM* for volume fractions around 0.3. Such a value corresponds to an infinite *fcc* lattice and is far less than the initial value measured experimentally for the initial stage when Bragg is first detected (*FWHM* ≈ 4%). This narrowing down occurs during the first few hours in agreement with the well-known finite size effects [42], that cause a *FWHM* decrease as thickness increases from a few layers to a few tens where the crystal reaches a thickness that can be considered infinite and where optical features remain stationary, accordingly. Growth beyond this thickness is detected with no apparent changes in the reflectance spectrum.

**Figure 5:**
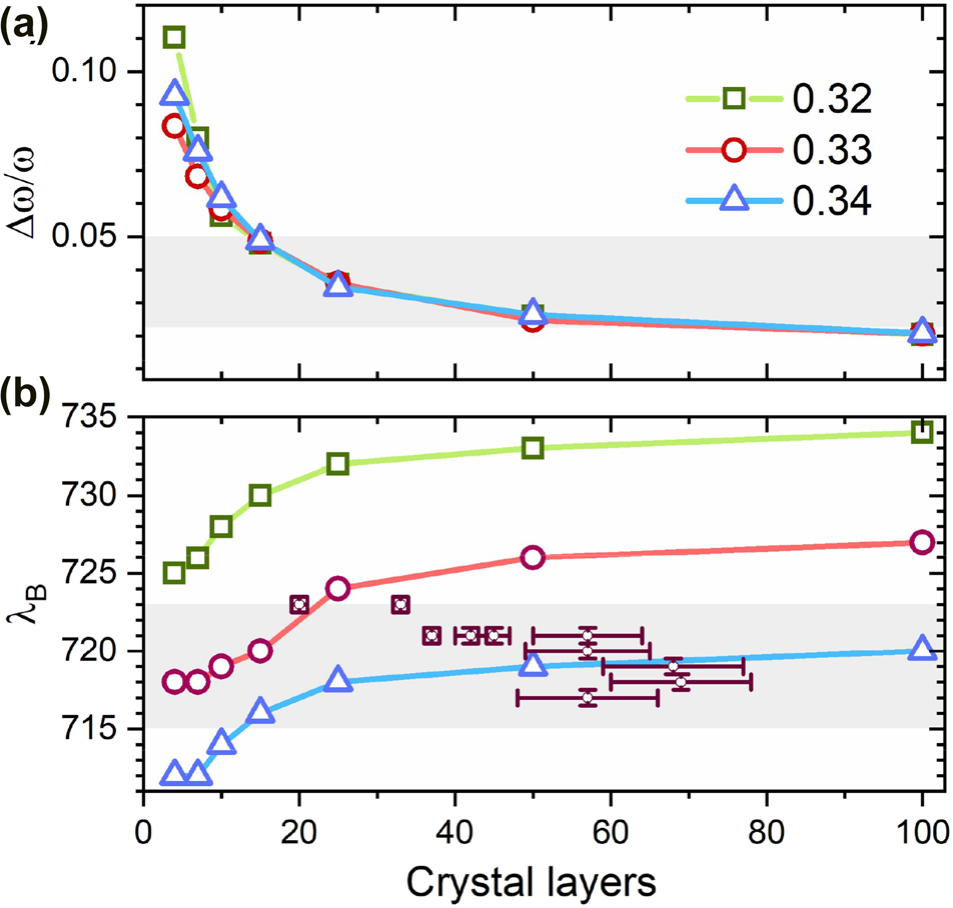
Calculation of FWHMs (a) and Bragg peak positions (b) as a function of the number of crystal layers (b) by SWA. Experimental FWHMs determined the number of crystal layers with different three different filling fractions. These experimental values (solid squares) are incorporated in (b) to determine the compatible filling fractions.

The simultaneous increase in thickness and in volume fraction have opposite effects on Bragg wavelength, the former tending to increase and the latter to decrease it. However, the effect of the former is more significant in the reduction of the *FWHM* (see Figure 5a) while the latter mainly affects the shift of the Bragg wavelength: more than 5 nm for each hundredth of a volume fraction (Figure 5b). Therefore, the reduction in the *FWHM* observed during stage II can be attributed almost exclusively to an increase in the thickness of the crystals whose effect is saturated after a few tens of crystal layers. This is shown in Figure 5a where the evolution of *FWHM* (Δ*ω/ω*) represented as a function of thickness in crystal layers obtained by a calculation using the SWA for three different filling fractions. Figure 5a shows that, for the measured spectra (*FWHM* < 5%) crystal thickness can be estimated, regardless of *ϕ*, from the experimental peak widths. These values and their corresponding wavelengths (from Figure 4) can then be plotted in Figure 5b along with the SWA numerical estimates. This comparison shows that as the crystal grows volume fraction takes on values that grow from barely below 0.33 to slightly above 0.34 as thickness grows from about 22 crystal layers at the beginning of stage II to about 70 crystal layers at the end. In other words, during this stage, the structure grows in thickness with a slight compaction. Here, it is very important to emphasize the fact that the initial estimated value of thickness, 22 crystal layers for the beginning of this stage, is only determined by the first measurement made and is critically dependent on early detection: if the peak rises over the background a little earlier (thinner crystal) or a little later (thicker crystal) depends on the ability to detect a small peak on a few percent reflectance background. To the best of our knowledge, access to the first layers of growth had only been possible through confocal microscopy [1]. Our approach is much simpler, it offers spectroscopic information and is able to follow this process to the very end of the drying stage.

The formation of the Bragg peak with large spheres, *d* = 377 nm, is similar to that shown in Figure 3b, although in this case, higher energy bands are revealed in this wavelength range. Here optical response provides information about the lateral assembly of the structure, forming and narrowing as the thickness of the crystal grows (Figure S5) [43]. At the same time that the Bragg peak narrows its intensity increases until saturation. That the background does not change, and the peak soon saturates suggests that crystallites originating diffraction are not obscured by (diffuse) Mie scattering from fluid, implying that they grow closer to the light incidence.

**Figure 6:**
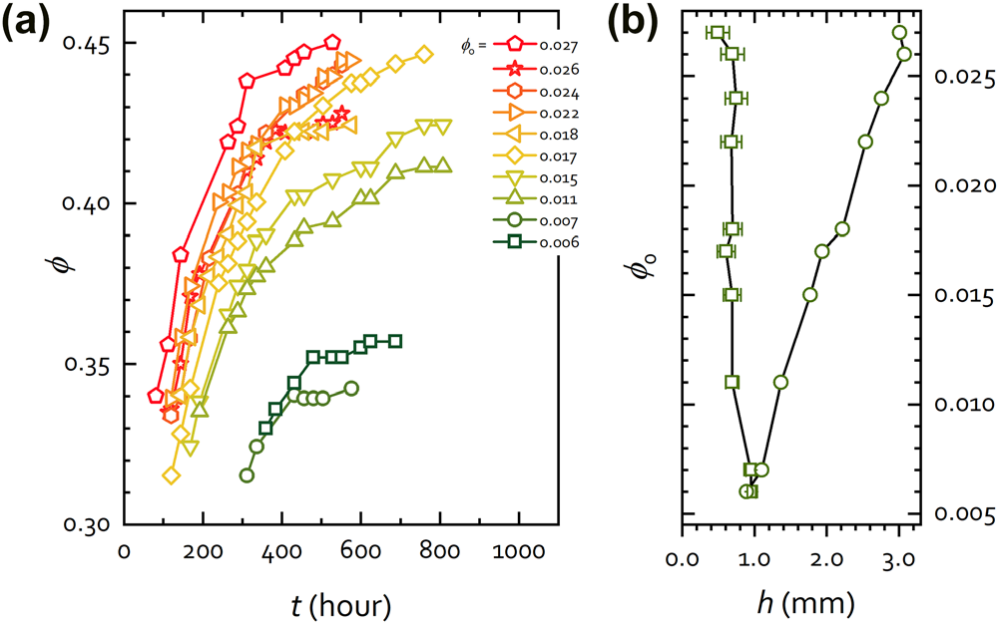
Crystal growth in region III. (a) Crystal volume fraction *ϕ* as a function of time extracted from Eq. (1) for various initial colloidal suspensions characterized by their initial concentration *ϕ*_0_. (b) Equivalent thickness as a function of *ϕ*_0_ calculated with Eq. (2) from the initial (square symbols) and the final spectra (round symbols). The crystallization of the colloid is produced when approximately equal equivalent thicknesses are reached regardless of the initial volume fraction of the suspension.

All these facts sustain the conjecture that the crystalline phase grows from the substrate upwards into the fluid as would otherwise be natural from their higher density. At the end of this stage, crystals are grown that present volume fractions between 0.31 and 0.34 (see Figure 6a, first data of each *ϕ*_0_ set).

### 3.3 Crystal compaction *(131–800 h)*

After the initial period of fast intensity growth (II) that serves to define the peak, it further strengthens (at a slacker pace) and shifts towards shorter wavelengths for several hundred hours.

This, initially fast, blue shift of the peak is related to the packing of the structure against the substrate. As the natural sedimentation progresses, the settled spheres gain accessible volume which results in a progressively more close-packed structure, increasing the filling fraction, *ϕ.* In other words, the periodicity of the lattice decreases and the average refractive index grows continuously as more spheres settle, causing the blue shift of the band gap. This blueshift slows down and, at longer sedimentation times, comes to a stop; Bragg wavelength stabilizes indicating the end of the sedimentation process (Figure 1c). This is shown in Figure 4, region III. The final position of the Bragg peak depends on the initial volume fraction, that is, the actual number of particles involved, and it is reached very slowly (hundreds of hours). At this point, depending on their initial colloidal volume, the crystals typically present volume fractions between 0.36 and 0.45 (Figure 6a). Crystal growth and its accompanying shrinking is revealed also by an increase in reflectance caused by the growing volume contributing to Bragg reflection (see reflectance in Figure 4, region III). The compaction of the crystals can be described using the theory of photonic bands calculated by MPB [44]. This is shown in Figure S6 where the evolution of the theoretical bands is remarkably consistent with the shift of the Bragg peak. From the above, it follows that the thickness of the crystal formed in region III is large enough to be considered infinite and described by bands theory.

Between the onset of crystallization and the final crystal configuration the volume fraction grows as depicted in Figure 6a. For each initial volume fraction, *ϕ*_0_, the first data point indicates the formation of the crystal (time indicating threshold thickness; wavelength, initial volume fraction) and the last facilitates the final thickness (time) and volume fraction (wavelength). For a better presentation the data set labels on this figure have been made to match the ordinate axis in Figure 6b that represents the height of the grown crystal in the sediment as a function of *ϕ*_0_. From Figure 6a one can see how as the initial volume fraction grows so do the crystal volume fractions, resulting in the curves shifting upwards. Because the spectral features of the incipient Bragg peak are intrinsically uncertain it is difficult to provide accurate values for the first volume fractions calculated from the reflectance data. Besides, the onset of Bragg diffraction can be used to determine the threshold thickness as in Figure 6b. Not surprisingly, the threshold values for this parameter—minimum deposit thickness that gives rise to the first hints of a Bragg peak—are similar for all initial concentrations.

As the sedimentation of the spheres progresses, a volume of clear water appears at the top of the vessel whose interface with the colloidal suspension moves with a velocity *U* (*ϕ*_0_) according to modified Stokes’ law whose expression is: *U* (*ϕ*_0_) = *U*_0_ (*ϕ*_0_) *K* (*ϕ*_0_) were *K* (*ϕ*_0_) = (1 − *ϕ*_0_)^*n*^ with *n* ≈ 6.6 [45]. The spheres depleted from this volume accumulate at the bottom as a sediment in which the colloidal fluid and crystal may coexist. Because the particle number is constant, the volume times the initial volume fraction is a constant across the phase change and the thickness of the sediment, *h* (*t*), can be worked out from:

(3) $$h\left(t\right)A\phi =tU\left(\phi_{0}\right)A\phi_{0}$$

where *t* × *U* (*ϕ*_0_) is the height of depleted volume; *ϕ*_0_, the initial volume fraction of the prepared colloid; *ϕ*, that of the sediment and *A*, the area of the vessel. By recording the depleted volume (height or time) when the first hint of a Bragg peak is detected a threshold thickness can be established for a crystal to form. Conversely the total height of the depleted water (or time) provides the final thickness of the crystal deposited for which the plane spacing and the crystal layer thickness can be calculated (see SI and Equation S1).

Square symbols in Figure 6b represent the threshold thickness required for each initial concentration (*ϕ*_0_) to show the first hints of the Bragg peak assuming that the deposit has the density of the crystal being formed. Since the fluid has a lower volume fraction, it will have a larger height and, therefore, the estimated thickness is a lower bound to the actual sediment thickness. This threshold is almost constant for the sphere sizes and concentrations studied. Therefore, the transition from a colloidal fluid to a crystal, marked by the formation of the band gap, is independent of the initial volume fraction as long as it is in the favorable range.

### 3.4 Water extraction *(800–1000 h)*

Once the sedimentation of the colloid is accomplished, the natural evaporation of the spheres-depleted supernatant water above the crystal would take many days, reason for which we extract it by pipetting. This is done carefully minimizing the perturbation of the process. Nonetheless, after some unavoidable perturbation, a transient period (some 200 h) ensues where the equilibrium reached is disrupted and restored. It is worth noting here that, once the crystal is formed and stable, perturbations produced by, for instance, stirring the water or knocking the cylinder, if gently done, result in a slow redshift of the Bragg peak that is eventually mitigated, the peak slowly recovering its equilibrium position and spectral characteristics in a matter of a few hours.

From then on, the rest of the water removal will be left to natural evaporation under ambient conditions without much change to the crystal as shown in Figure 4, region IV.

### 3.5 Compression of fluid by evaporation *(1000–1017 h)*

After some additional idle hours, evaporation of clear water brings the air-water interface to the top of the sediment. At this stage, the last spheres belonging to the colloidal fluid phase are deposited on the sediment, causing the freezing of the structure (*ϕ* ∼ 0.49). Any further evaporation will limit the volume available to the colloidal fluid, further compressing and reducing the lattice parameter, increasing the volume fraction and causing the Bragg peak to shift towards shorter wavelengths (*λ* in Figure 4, region V). At this point, the gradient in the concentration profile of spheres as a function of height disappears, because the volume provided by water follows the same course, and the spheres of the upper layers are incorporated into the sediment. The latter causes *ϕ* to increase and reach the value of the melting point, *ϕ* ∼ 0.54, so the crystallization of the whole colloid is completed. The melting marks the appearance of multiple defects in the crystal [46], such as dislocations and domain boundaries since size polydispersity is no longer accommodated by the medium, as is evidenced in a dramatic decrease in intensity (See *R* in Figure 4, region V) due to loss of crystallinity of the sediment.

### 3.6 Compression of the crystal *(1017–1031 h)*

Until this moment the crystal had a filling fraction lower than that at the melting point, allowing equilibrium with the fluid preserving the highest quality possible since its crystallinity was granted by colloidal equilibrium, setting a lattice parameter larger than that corresponding to close packing (with a plane spacing of 204 nm). Now, spheres are forced to further pack, to a point where their arrangement is governed by their size. At this stage, crystal quality is determined by sphere polydispersity which is evidenced by a decrease in intensity (See *R* in Figure 4, region VI). At this point, the crystal presents a volume fraction, *ϕ* ∼ 0.7, according to the optical data.

Since there are four spheres per unit cell in the *fcc* lattice the filling fraction is *ϕ =* 4*V*_s_ *a* ^−3^ where *V*_s_ is the volume of a colloidal particle. This yields Δ*ϕ*/*ϕ* = −3 d*a*/*a* which, from Eq. (2) gives Δ*a*/*a* = 1.1 Δ*λ*/*λ*. This is a remarkable result in that nanometer precision of the Bragg wavelength detection (0.2% uncertainty) results in sub-nanometer resolution in the corresponding lattice parameter.

### 3.7 Drying of the self-assembled crystal *(1031–1037 h)*

Once close packing is reached, any further removal of water can only be achieved by exchanging it for air. This marks the beginning of the evaporation of the water *within* the structure with very limited lattice parameter shrinkage [47]. This exchange tends to reduce the average refractive index (hence the Bragg wavelength) and increase the dielectric contrast which accounts for the peak broadening. An increase of the background in the spectra is also accounted for by the stronger scattering customary in dry opals. Naturally, after all the water has evaporated, the volume fraction of the *fcc* lattice approaches, but never reaches *ϕ* = 0.74 [48]. This is due to imperfections such as vacancies, stacking faults, domain boundaries, etc., which on average reduce the filling fraction but more importantly due the fact that, at room temperature, some interstitial water still remains forming “necks” between the spheres [27, 49]. Only forced drying by heating can fully deplete the resulting opals of water and restore the close packed filling fraction. The resulting material in region VII is a typical partially moist opal.

### 3.8 End of drying *(>1037 h)*

The absence of any variation in the optical parameters in this region indicates that the drying process is complete and therefore the opal has finished its assembly.

## 4 Conclusions

Real-time, *in situ* exploitation of the photonic crystal properties of self-assembled structures hands in the evolution of the lattice parameter—hence, volume fraction—and crystal size of the assembling structure as a function of time. The technique provides sub-nanometer resolution in the lattice parameter of the colloid during crystallization and drying.

The crystallization process is found to originate close to the substrate, adding layers and growing towards the top of the sediment in agreement with previous observations [1, 15]. The organization of the spheres was tracked during the evaporation of the background medium revealing three successive processes. First, the seeding of the crystal at the expense of colloidal particles settling at Stokes’ velocity is registered. Second, the packing of the structure as the surrounding water evaporates. And lastly, a decrease in the effective refractive index and the increase of dielectric contrast is found, caused by the water replacement with air during drying, much like that obtained in *ex situ* experiments [25, 50, 51].

Finally, this technique can be extended to different materials in order to elucidate how the first layers of particles self-assemble and their subsequent growth as more material is added to the crystal. More complex colloids such as binary or even ternary mixtures [52, 53] could, after careful selection of particles size and proportion, permit to witness crystallization into multiple strata. Modification of the setup by observing from above could improve the potential applicability. This new approach can enable the crystallization of micrometric spheres that have multiple applications in various fields [54–58].

## Supplementary Material

Supplementary Material Details
