# Supplementary material for: Colloidal photonic crystals formation studied by real-time light diffraction
Source: Nanophotonics. 2022 Jun 9;11(14):3257–67. doi: 10.1515/nanoph-2022-0127 (PMC11501291; doi:10.1515/nanoph-2022-0127)
Supplement: Supplementary file 1 — Supplementary Material Details [file j_nanoph-2022-0127_suppl.pdf]

Supplementary Information for:

Colloidal photonic crystals formation studied by real-time light diffraction

*Jose Ángel Pariente, *Álvaro Blanco and Cefe López*

Instituto de Ciencia de Materiales de Madrid (ICMM); Consejo Superior de Investigaciones Científicas (CSIC) Calle Sor Juana Inés de la Cruz 3, E-28049 Madrid, Spain

Email: j.pariente@csic.es

Experimental setup

Figure S1 shows the experimental setup used to monitor the optical spectra taken from the underneath of the samples. A standard halogen lamp is used as the light source focused onto an optical fiber. The beam splitter is the key part of this setup as it splits the light beam in two halves. One of these halves is dumped while the other, collected by another optical fiber, is incident on the sample with a collimated beam of 2 mm diameter. The reflected light is collected at normal incidence by the same fiber. This light returns to the beam splitter and from there to an Ocean Optics USB2000+ spectrometer operating in the visible range. Real-time diffraction of the light allows characterization of particle assembly during the natural settling process of silica spheres.

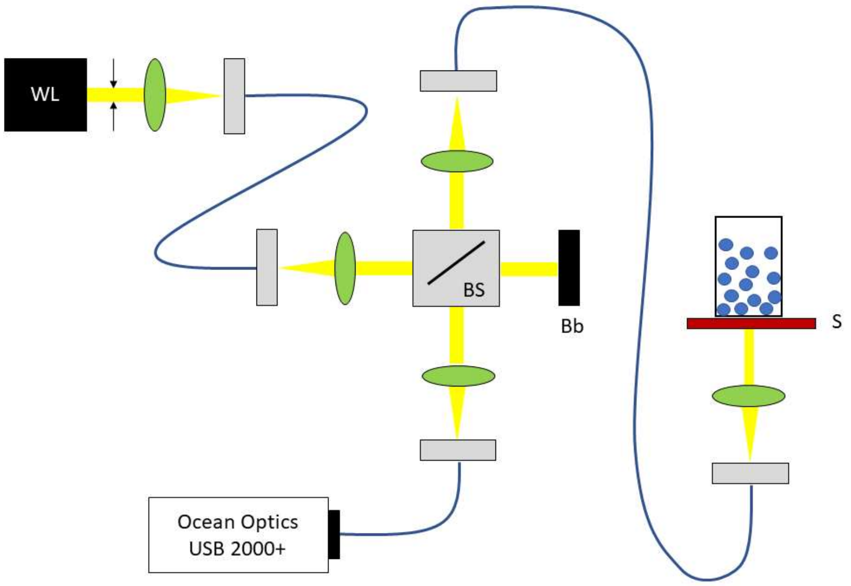

Figure S1. Schematic illustration of the experimental setup used for natural sedimentation monitoring. The optical fibers are drawn in blue.

Real-time Bragg peak evolution

The crystallization of the colloidal suspension is monitored by the evolution of the Bragg peak. Real-time light diffraction enables this study as it can be seen in Figure 2. To clarify this behavior, Figure S2 shows different regions. Specifically, the colloidal suspension before the

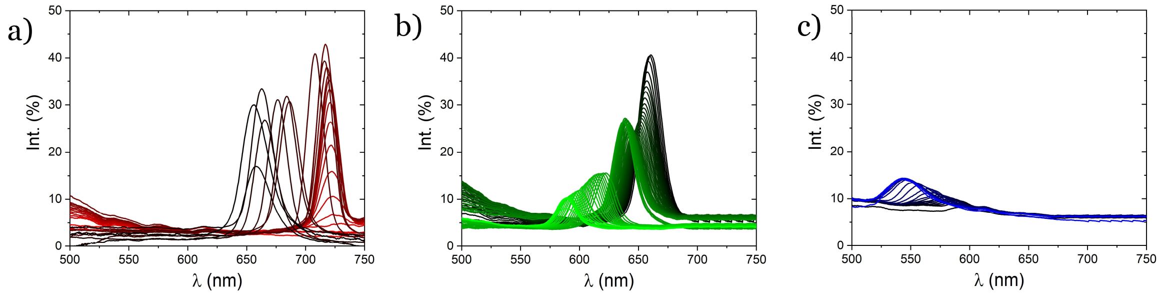

Figure S2. Real-time specular reflectance of the sample of Figure 2 split in different regions, before the evaporation process (a), until the air-water interface reaches crystal edge (b), and in the interstitial evaporation (c).

evaporation process (Regions I-III), Figure S2a, until the air-water interface reaches crystal edge (Regions IV-VI), Figure S2b, and in the interstitial evaporation (Regions VII-VIII), Figure S2c. Understanding this behavior is main goal of this work and is deeply described in Figure 4 and Table 1.

Crystallization of a colloidal fluid

In order to verify if a colloidal fluid can crystallize under certain conditions, a natural sedimentation of silica spheres with $\phi_0 = 0.005$ is monitored for 550 hours (the sedimentation is completed at 385 hours). This is shown in Figure S3 where no optical changes can be distinguished during these hours. At $t = 599$ hours (thick black line), a supplementary $V = 0.3$ mL ($\phi_A = 0.011$) of solution of the same spheres is added to the colloid resulting in an equivalent volume fraction

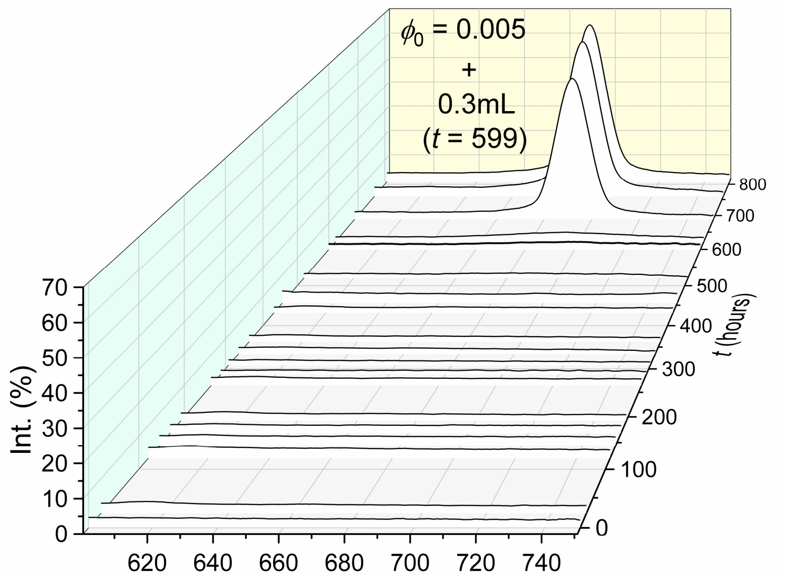

Figure S3. Real-time specular reflectance of a sample with an initial concentration under the threshold, $\phi_0 = 0.005$, to which a $V = 0.3$ mL ($\phi_A = 0.011$) of colloid is added. Once they settle, the addiction of these spheres forces the transition of the sediment to the crystalline phase.

of $\phi_E = 0.006$, thus above the threshold for crystallization. In just a few hours, the newly added spheres settle and cause the colloidal fluid deposited at the bottom to crystallize, as marked by the appearance of the Bragg peak as seen in Figure S3. The aforementioned causes the spheres located in the fluid phase to reduce their exclusion volume in order to increase the entropy of the system with the consequent transition to the crystalline phase.

Crystal nucleation

Under the same conditions, the initial volume fraction determines the time at which the suspension crystallizes in the natural sedimentation of silica spheres. This is shown in Figure S4 where the time of crystal nucleation is represented as a function of the initial volume fraction. Note that this time of nucleation is the first in which the Bragg peak is visible in each case and signals the time of transition from the colloidal fluid to the crystal. This time of nucleation is greater for smaller

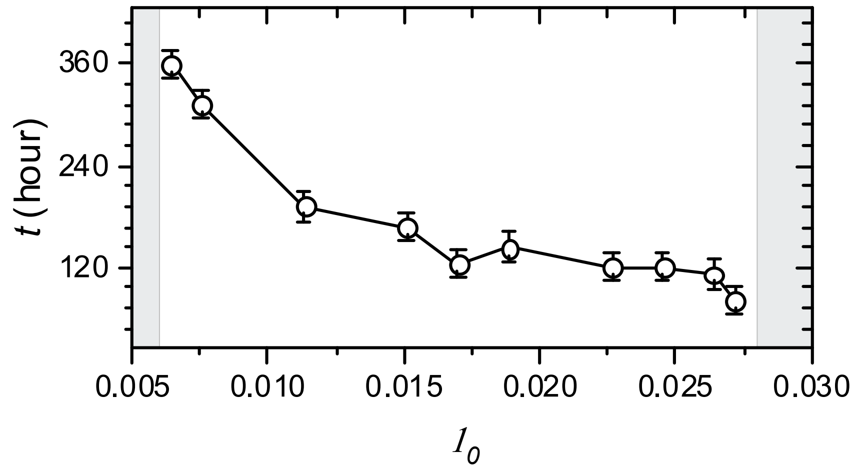

Figure S4. Time of crystal nucleation and growth initiation as a function of initial volume fraction. The earliest time at which a Bragg peak is visible marks the time when a crystal starts to grow (left edge of region II in Figure 4). The limits of the initial concertation range for crystal formation are marked by shaded areas.

initial volume fractions. This can be understood as the higher the initial colloidal concentration the sooner sufficient spheres reach the bottom just because more spheres are close to it.

Light diffraction of silica spheres with *d* = 377 nm

Natural sedimentation of silica spheres with $d = 377$ nm is monitored by light diffraction to obtain the band gap in the high energy region. The behavior of this sphere size is qualitatively the same, except for the actual ϕ_0 boundaries. The diffraction of the light is collected at the bottom as is shown in Figure S5 where the formation of this peak is monitored as a function of time. The formation, growth and evolution of this peak provides information about the lateral direction of the structure where the crystallization also takes place.

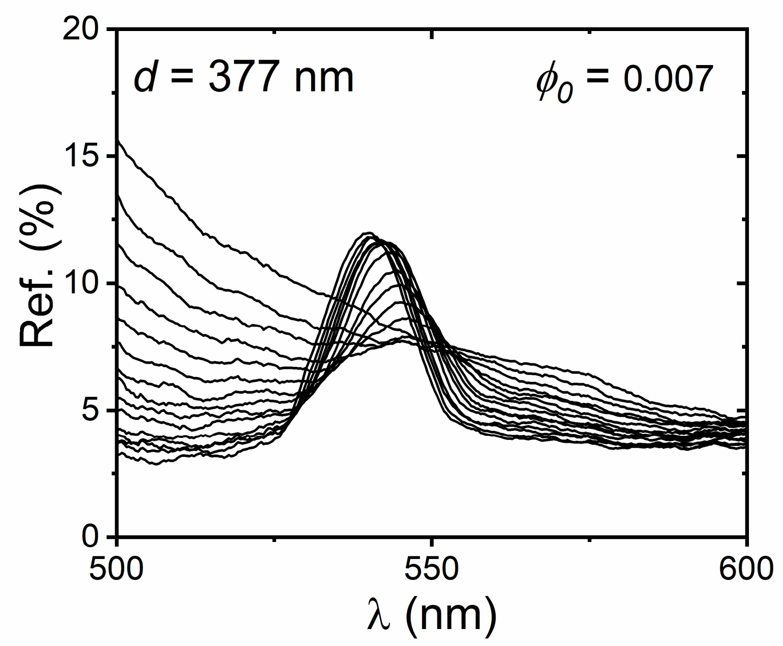

Figure S5. Crystallization of a natural sedimentation of silica spheres with d = 377 nm. The formation of the high energy peak indicates the lateral formation of the crystal.

Evolution of the photonic bands using the MIT photonic bands software

Theoretical calculations of the band structures were performed to check whether the behaviour described in the main text is consistent with the evolution of the bands when the structure is packed. *MIT Photonic Bands*, MPB, is a freeware package used to determine the theoretical bands. This software calculates the eigenstates of the Maxwell's equations using a planewave basis expansion.[2] The hexagonal lattice of a *fcc* in a medium with the same dielectric constant as water is defined using the $a_{eff} = \left(\frac{2\pi}{3\phi}\right)^{1/3} d$, thus different theoretical calculations of band structures are performed changing ϕ. Figure S6 shows the evolution of the band gap, for $d = 250$ nm, as a function of ϕ for different concentrations. As expected, high concentration samples evolve into

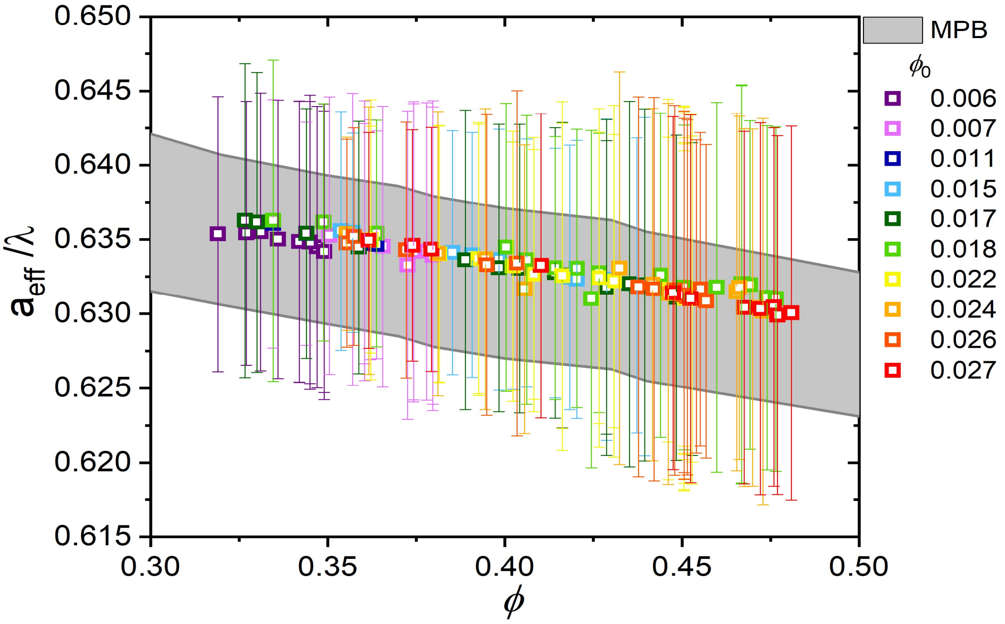

Figure S6. Evolution of the theoretical photonic bands simulated using MPB, grey shaded area, and the shift of the Bragg peak monitored for different initial volume fractions.

dense packing structures. The agreement between the values of ϕ obtained by the Bragg's law, colour dots, and the theoretical bands calculated by MPB, grey shaded area, is excellent. From the above, it follows that the thickness of the crystal formed in region III is so large that it can be considered infinite and described using MPB simulations.

Plane spacing of an *fcc*

The (111) plane spacing in an *fcc* lattice giving rise to the Bragg peak recorded, can be expressed as a function of volume fraction as:

$$d_{111} \approx 0.74 d / \sqrt[3]{\phi} \qquad \text{(S1)}$$

where d is the spheres diameter. It is worth noticing that the thickness of the initial sediment is about 0.7 mm for all crystallizing suspensions. In light of the volume fractions registered at nucleation —presented in Figure 6— that fall between 0.31 and 0.33, the plane spacing ranges between 260 and 267 nm and therefore the sediment is about 2700 crystal layers thick. Of these, only a few are ordered when the Bragg peak appears and subsequently the sediment is compacted by the gain in accessible volume, as seen in the discussion of stage II. Once all the spheres have been deposited, the final thickness, Figure 6b circles, will naturally be linearly dependent on the initial volume fraction of spheres (number of particles to crystallize). At this stage (crystals compacted) the filling fraction indicates that the crystal presents a plane spacing of around 240 nm.

References:

[1] C. López, L. Vázquez, F. Meseguer, R. Mayoral, M. Ocaña, and H. Míguez, "Photonic crystal made by close packing SiO_2 submicron spheres," *Superlattices Microstruct.*, vol. 22, no. 3, pp. 399–404, 1997.

[2] S. Johnson and J. D. Joannopoulos, "MIT Photonic-Bands," 2017. [Online]. Available: https://mpb.readthedocs.io/en/latest/.
